# Local Shearing Force Measurement during Frictional Sliding Using Fluorogenic Mechanophores

**DOI:** 10.1021/acs.jpclett.2c02010

**Published:** 2022-09-16

**Authors:** Chao-Chun Hsu, Feng-Chun Hsia, Bart Weber, Matthijn B. de Rooij, Daniel Bonn, Albert M. Brouwer

**Affiliations:** † van’t Hoff Institute for Molecular Sciences, University of Amsterdam, Science Park 904, 1098 XH Amsterdam, The Netherlands; ‡ Advanced Research Center for Nanolithography, Science Park 106, 1098 XG Amsterdam, The Netherlands; § van der Waals-Zeeman Institute, Institute of Physics, University of Amsterdam, Science Park 904, 1098 XH Amsterdam, The Netherlands; ∥ Laboratory for Surface Technology and Tribology, Department of Engineering Technology, University of Twente, P.O. Box 217, 7500 AE Enschede, The Netherlands

## Abstract

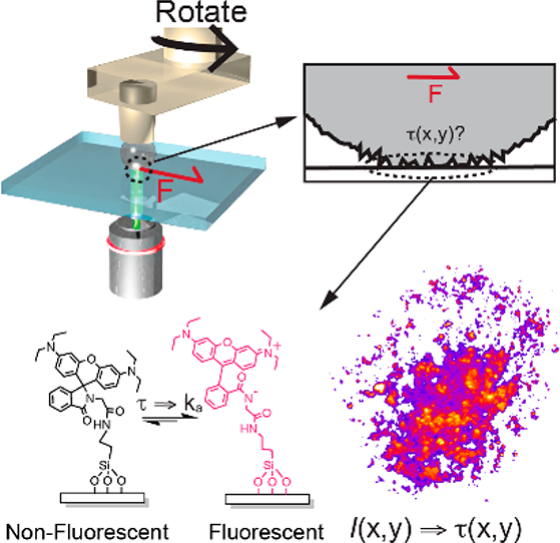

When two macroscopic objects touch, the real contact typically consists of multiple surface asperities that are deformed under the pressure that holds the objects together. Application of a shear force makes the objects slide along each other, breaking the initial contacts. To investigate how the microscopic shear force at the asperity level evolves during the transition from static to dynamic friction, we apply a fluorogenic mechanophore to visualize and quantify the local interfacial shear force. When a contact is broken, the shear force is released and the molecules return to their dark state, allowing us to dynamically observe the evolution of the shear force at the sliding contacts. We find that the macroscopic coefficient of friction describes the microscopic friction well, and that slip propagates from the edge toward the center of the macroscopic contact area before sliding occurs. This allows for a local understanding of how surfaces start to slide.

When static friction changes to dynamic friction, accidents may happen: we fall, our car crashes, or the earth trembles, just to name a few, all with detrimental consequences. Because of this, it is important to know how and when two surfaces start to slide with respect to each other.^1,2^ Answering this question is difficult because it is the real area of contact between the surfaces that determines the frictional behavior, and these are always rough over a broad range of length scales. This makes it challenging to describe the contact mechanics, i.e., where, and how much, precisely the two rough surfaces touch.^3^ Even if this is achieved, it is unclear when the surfaces in contact will begin to slide; frictional interfaces may even slip at some locations and stick in others, leading to unstable stick–slip friction initiated by rupture fronts that travel across the interface prior to overall sliding.^4−6^ The macroscopic friction coefficient is just an average ratio between normal stress and frictional shear stress; the central question is then how to go from the single asperity scale to the many-interacting-asperities macroscopic scale.

Single asperity friction experiments suggest that low-normal stress contacts are more easily sheared than contacts that experience a high normal stress.^7,8^ implying that indeed some parts of the frictional interface may begin to slide before others do. If this is true, this provides the key to understanding how and when surfaces begin to slide, provided one has a means of obtaining the local normal stress. It is in addition experimentally extremely challenging to observe very small displacements involved in local sliding events at a multiasperity interface.^9−11^ Here, we take a radically different approach and set out to directly measure the local frictional stresses. We reveal a new method for directly mapping out the shear stress in a (pre)sliding interface using a shear sensitive fluorescent mechanophore attached to one of the two surfaces. The mechanophore fluoresces when subject to stress and stops fluorescing when the stress is relieved: the stress causes a structural change in the molecules that makes them fluoresce upon excitation with visible light.^12−15^

As the mechanophore, we use rhodamine spirolactam RhGly (Figure 1).^16−20^ The molecules act as mechanophores because of their weak N–C bond, which permits ring opening due to an applied stress.^14,21^ We chemically link RhGly onto glass coverslips; by mapping out the position-dependent fluorescence intensity with fluorescence microscopy, we can visualize simultaneously the area of contact and shear stress.

**Figure 1:**
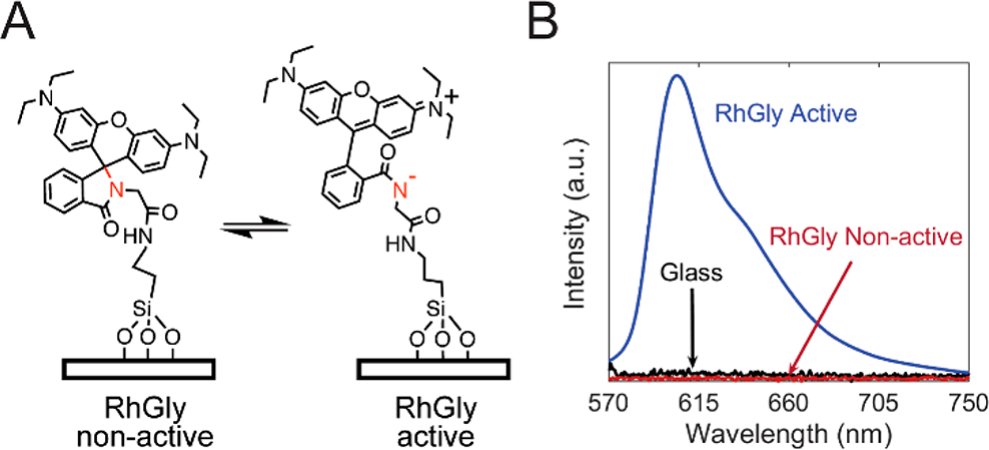
Working principle and spectral properties of the immobilized mechanophore RhGly. (A) Structure of RhGly immobilized at the glass surface, in the non-active spirolactam state, and in the shear-activated rhodamine form. (B) Emission spectra of immobilized RhGly (λ_exc_ = 560 nm). The activated form is generated here by adding acetic acid. Removal of the acid reverses the process (Figure S1).

The friction experiments are carried out using a total internal reflection fluorescence microscope, with a rheometer mounted on the microscope (Figure S5, inset). A (poly)methyl methacrylate (PMMA) or polystyrene (PS) bead is mounted off-center on the rheometer tool so that when the tool rotates, the bead slides over and activates molecules at the coverslip with a sliding speed of 100 nm/s while the macroscopic shear and normal force are recorded simultaneously (Figure 2A). We find that the ring opening induced by the shear force converts the dye to its fluorescent ON state. When the shear force is removed, the probe returns to the non-emissive OFF state with a lifetime of ∼230 ms (Figure S9),^17^ enabling the use of the method for dynamic friction measurements. The inverted microscope allows us to dynamically visualize the stresses as well as the real contact area through the fluorescence of the activated molecules (Figure 2B). The contact areas can be determined by counting the contact pixels after Otsu thresholding of the fluorescence image.^22−25^

**Figure 2:**
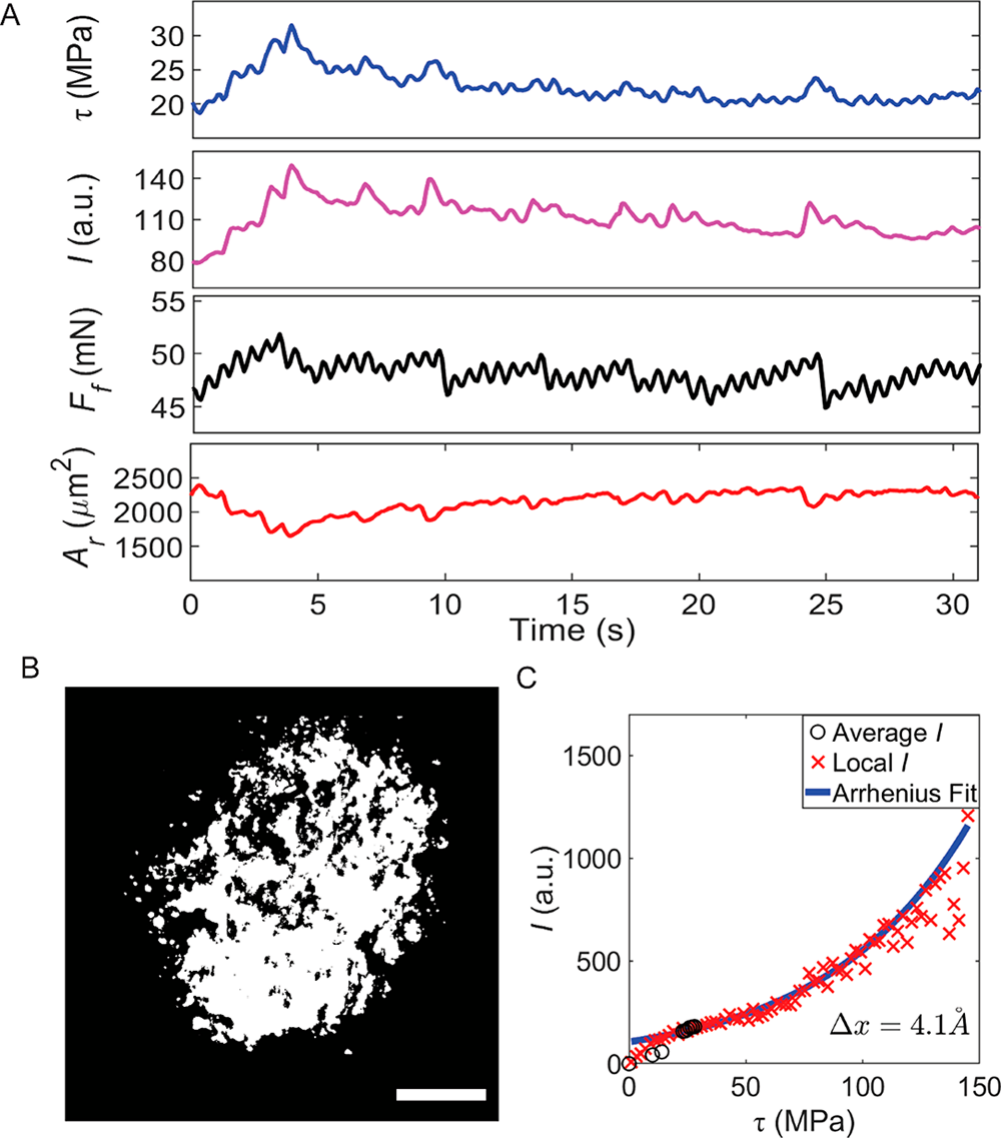
Sliding of a polymer bead over a RhGly interface. (A) Friction force *F*_f_, real contact area *A*_r_, shear stress τ = *F*_f_/*A*_r_, and fluorescence intensity *I* as a function of time during a typical sliding experiment (nonlubricated contact). Movie S1 shows a time series of the intensity images. (B) Representative fluorescence image during a typical sliding event, binarized with the Otsu threshold method to determine *A*_r_. Scale bar of 20 μm.^22−25^ (C) Fluorescence intensity *I* (black circles) as a function of shear stress (within contact during sliding, i.e., from 6 to 32 s in panel A). The local *I* vs shear stress data (red crosses) are acquired by superimposing the fluorescence image and simulated tangential stress (see Figure 3A) as described in the text. The two intensity data sets are fitted with Arrhenius behavior (eq 1, blue line). The activation length is found to be 4.1 Å.

To calibrate the probe, we quantify the overall fluorescence intensity as a function of the applied average shear stress. We observe that in a typical sliding experiment (Figure 2 and Movie S1), the fluorescence intensity as a function of time correlates well with average shear stress τ at the interface as calculated by τ = *F*_f_/*A*_r_, where *F*_f_ is the friction force and *A*_r_ the area of contact derived from the fluorescence pattern. The contact area shrinks before the onset of sliding, in agreement with previous reports.^25,26^ Fluorescence intensity *I* can be described theoretically by assuming that without stress, the reaction rate for the interconversion between the nonfluorescent and fluorescent forms is given by an activated process. The stress biases the molecules toward the fluorescent state, decreasing the effective activation energy for turning on fluorescence. Indeed, we find that the data in Figure 2C can be described well by an Eyring-type equation that takes the lowering of the reaction energy barrier by the shear stress into account (eq 1).^27,28^
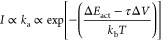
1where *I* is the fluorescence intensity, *k*_a_ and Δ*E*_act_ are the rate constant of isomerization and the activation energy of the molecule’s transition from the non-active to active form in the absence of force, respectively,^28^ and *ΔV* is the activation volume, i.e., the molecular volume difference between the non-active form and transition state of the mechanophore, with a corresponding length scale usually on the order of several angstroms.^29,30^ To extend the range of the measurements, the experiment is also performed with isopropanol and silicon oil as lubricants, giving rise to different friction coefficients (Figure S2). This can safely be done because the intrinsic fluorescence quantum efficiency is only weakly dependent on the lubricant, as indicated by the small differences in fluorescence lifetimes for different lubricants (Figure S3). We find that eq 1 can be used to describe the macroscopic relation (black dots in Figure 2C) between fluorescence intensity and shear stress.

It is noteworthy that the contact area can be visualized in a static experiment, without sliding. The reason is that the deformation of the asperities upon application of normal force causes local shear stress at the contacts. To demonstrate this, we perform static contact experiments with the lubricants mentioned above (Figure S3). The contact area is the same in all three cases. The fluorescence intensity, however, correlates with the lubricating ability because the lubricants reduce the shear stress at the contact. This shows that the fluorescence intensity during the frictional event reflects the shear stress within the real contact area.

Figure 2A reveals that, on the macroscopic scale, the in-plane stress builds up roughly linearly over time within the contact without sliding, until at 3.9 s macroscopic sliding accompanied by shrinkage of the area occurs when the shear stress exceeds a critical value. To understand this critical value is the key to understanding many friction problems, but one needs to see what happens at the microscopic scale to meet this challenge. We therefore ask how the microscopic friction behavior is reflected in the macroscopic friction coefficient by looking at how the local shear and normal stresses balance on the microscopic scale, both experimentally and theoretically. To quantify the local stress theoretically, we infer it from the contact mechanics. We perform the corresponding experiments using a PMMA bead for which the surface roughness has been measured using an optical profilometer prior to the sliding (Figure S6). The measured surface profile allows the performance of a finite (boundary) element simulation that maps out the contact of the sphere with the glass surface. In the model, the linear elasticity (Table S1 and Figure S5) and a local maximum friction coefficient equal to the global friction coefficient are assumed.^11,31^ Under these assumptions, the contact area as well as the local normal and shear stresses within the area of real contact can be predicted as a function of the externally applied normal and tangential forces.

The model predictions of the contact area and local shear stress can then be compared to the experimental stress and contact area measurements. Figure 3A shows that the simulated stress distribution and the fluorescence image at the onset of sliding (at 32 s in Figure 4 and Figure S7) show a remarkable and quantitative similarity. In both images, a distribution of intensities is observed, showing the local variations of the shear stress. The correlation between the local intensity and the local shear stress can be obtained by comparing, pixel by pixel, the simulated shear stress to the experimental fluorescence intensity when superimposing the two 100 000 pixel images. The pixels are binned by shear stress per megapascal, and the corresponding fluorescence intensities in the contact points are averaged. Figure 2C shows the direct relation between the measured intensity and the local simulated tangential stress. Spectacularly, the Eyring relation (eq 1) also describes the local intensities with the same parameters as the global intensities.

**Figure 3:**
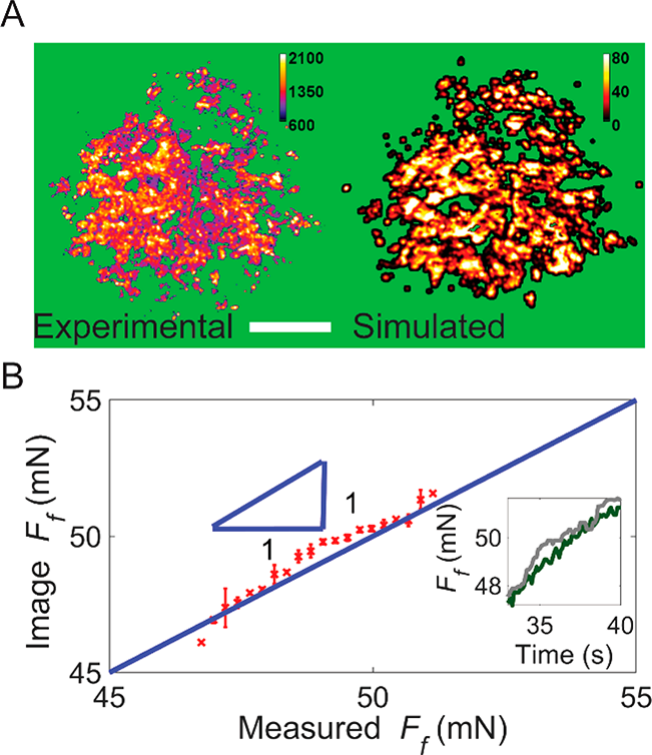
Local shear stress simulations vs experiments. (A) Experimental local shear stress τ (left) as obtained from fluorescence images (sliding speed of 100 nm/s). Simulation (right) using a boundary element model at the onset of sliding. Scale bar of 20 μm. The time evolution of the simulated local shear stress is shown in Movie S2. (B) Comparison of friction force *F*_f_ calculated from fluorescence images with the value obtained from independent rheometer data during the sliding. The solid blue line has a slope of 1. The inset shows force–time curves for both methods (gray, from fluorescence images; green, from rheometer data).

**Figure 4:**
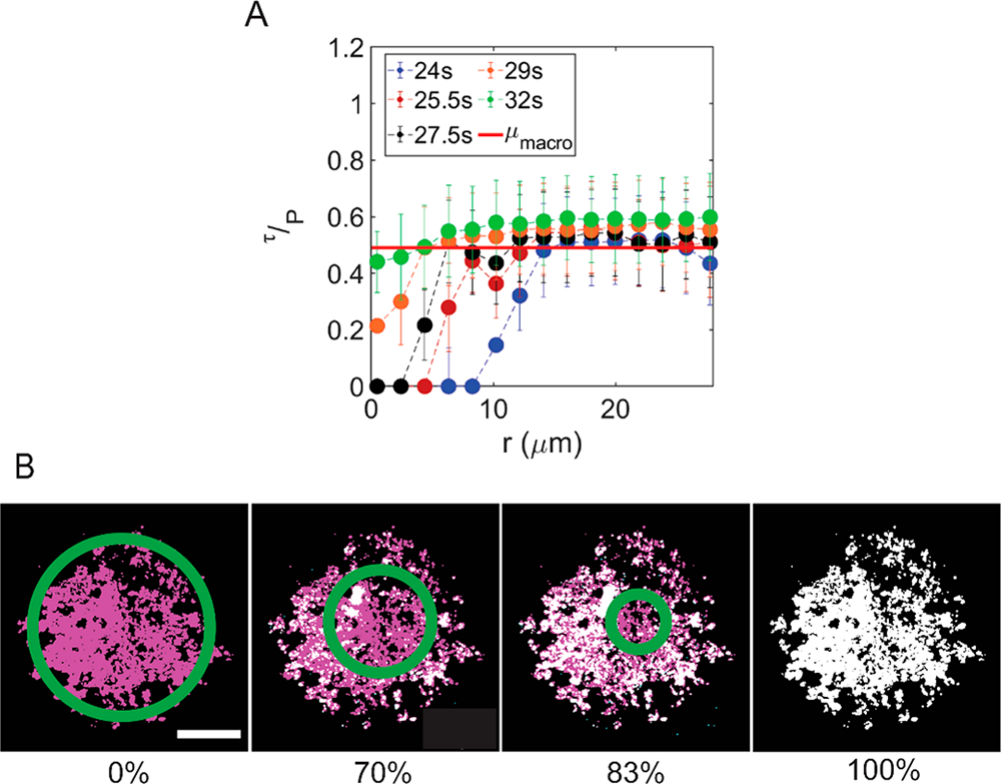
Evolution of the friction coefficient within the contact area before macroscopic slip. (A) Ratio of local shear stress to local normal pressure as a function of radial distance *r* to the contact center using the data of Figure 3A. The solid red line represents the macroscopic coefficient of friction. (B) Propagation of the slip zone (white) during presliding. Regions at the interface within which the ratio of local shear stress to local normal stress is equal to the macroscopic friction coefficient are colored white, and regions within which the ratio of shear stress to normal stress is smaller than the macroscopic friction coefficient are colored magenta. The percentages indicate what fraction of the interface has slipped. The green circles approximately enclose the stick zone. Scale bar of 20 μm. An animation of the transition is shown in Movie S3.

To demonstrate this, we show that we can now quantitatively describe the friction solely by the fluorescence image; to do so, we take the experimental shear stress distribution during sliding and calculate the overall friction force by integrating all of the local shear stresses within the contact area, i.e., ∫τ(*x*, *y*) d*A*. The results closely resemble the global friction force directly measured with the rheometer during the sliding experiment (Figure 3B and Movie S2).

The mapping, therefore, allows us to bridge the gap between the microscopic and macroscopic scales and to determine the criterion for the onset of sliding. The experiments show that local slip at the interface takes place if the local shear stress exceeds a critical value *μτ*(*x*, *y*) = *P*(*x*, *y*), because Coulomb’s friction law is valid locally. Quantitatively, we can calculate the local friction coefficient from the local experimental shear stress divided by the contact pressure from the boundary element model.

Arguably, the most important question this allows us to answer is how and when the sliding starts. Figure 4A shows the time evolution of τ/*p*, obtained by plotting the experimentally measured shear stress divided by the calculated normal stress, as a function of distance *r* to the contact center. As the system gets closer to macro-slipping, one can see the plateau value of the ratio moving toward the center (*r* = 0), until the microscopic friction coefficient equals the macroscopic value over the full contact area, at which point (*t* = 32 s in Figure S7) macroscopic slipping occurs. The onset of sliding is then given by the criterion that all contact points must have a shear stress exceeding the critical value as described by Mindlin’s solution (Figure S8).^6^ Before this happens, no sliding occurs. In the sphere-on-flat contact we probe here, the normal force is highest at the center of the contact and decays to zero at the edge of the contact. Because the local and global friction coefficients are identical, this means that the edges can already move before the center does. The experimental manifestation of this is shown in Figure 4B and Movie S3. A “wave” of slip propagates from the edge to the center, and the onset of macroscopic sliding happens when the wave reaches the center.

In conclusion, we show that a rhodamine spirolactam RhGly fluorescent probe can be used to map out *in situ* local shear stresses at the interface of two systems in contact prior to and during sliding. The molecules become fluorescent in response to shear stress, allowing us to study the real contact area and local shear stress between two surfaces in contact. We investigate Coulomb’s friction law by comparing the measured local stress to the local normal pressure obtained from a boundary element model, demonstrating the validity of Coulomb’s law at the local scale. Both local measurements agree with the measured macroscopic shear and normal forces. This allows us to experimentally visualize the evolution of the local shear and slip events before the onset of macroscopic slip. The measurements of the local tangential stresses in contact show that even though the shear stress and normal pressure are local variables, the ratio of these two perpendicular forces increases during preslipping to a maximum value equal to the macro-recorded coefficient of friction. These findings highlight the power of mechanophores to open an unprecedented “inside” view of the well-known phenomenon of friction between two objects and pave the way for a predictive and microscopic understanding of the onset of slip.

## Notes

All data are available in the main text or the Supporting Information.

## Supporting Information Available

Materials and Methods, Scheme S1, Table S1, and Figures S1–S10 (PDF)
Movie S1 (MP4)
Movie S2 (MP4)
Movie S3 (MP4)
