# Supplementary material for: Local Shearing Force Measurement during Frictional Sliding Using Fluorogenic Mechanophores
Source: J Phys Chem Lett. 2022 Sep 16;13(38):8840–4. doi: 10.1021/acs.jpclett.2c02010 (PMC9531245; doi:10.1021/acs.jpclett.2c02010)
Supplement: Supplementary file 1 — jz2c02010_si_001.pdf [file jz2c02010_si_001.pdf]

SUPPORTING INFORMATION

Local Shearing Force Measurement During Frictional Sliding Using Fluorogenic Mechanophores

Chao-Chun Hsu,[1] Feng-Chun Hsia,[2,3] Bart Weber,[2,3] Matthijn B. de Rooij,[4] Daniel Bonn, [3*] Albert M. Brouwer[1*]

[1]van 't Hoff Institute for Molecular Sciences, University of Amsterdam, Science Park 904, 1098 XH Amsterdam, The Netherlands

[2]Advanced Research Center for Nanolithography, Science Park 106, 1098 XG Amsterdam, The Netherlands

[3]van der Waals-Zeeman Institute, Institute of Physics, University of Amsterdam, Science Park 904, 1098 XH Amsterdam, The Netherlands

[4]Laboratory for Surface Technology and Tribology, Department of Engineering Technology, University of Twente, P.O. box 217, 7500 AE, Enschede, Netherlands

SUPPORTING INFORMATION

Table of Contents

Materials and Methods	S3
Supplementary Figures and Tables	S5
Supplementary Movies	S10
References	S10

Materials and Methods

In Scheme S1 we illustrate the functionalization procedure of RhGly at the coverslip. RhGly was synthesized according to the procedure in reference [1], and immobilized on glass microscopy cover slips. Clean coverslips were first silanized by adding immersing them in a mixture of 2 mL of APTES and 80 mL of 96% EtOH to which 2 mL of 99% acetic acid was added. The reaction was allowed to proceed for 30 mins at room temperature. The cover slips rinsed with absolute ethanol were annealed in an oven at 1 bar, 130 °C for 24 hrs. Silanized coverslips were placed in a reaction flask with RhGly (30 mg, 0.06 mmol), 1-[bis(dimethylamino)-methylene]-1H-1,2,3-triazolo[4,5b]pyridinium 3-oxid hexafluorophosphate (HATU) (46 mg, 0.12 mmol), and N,N-Diisopropylethylamine (DIPEA) (30 mg, 0.23 mmol). 65 mL of anhydrous dimethylformamide (DMF) was added. The immobilization was continued for 16 hours under argon at room temperature. The coverslips were cleaned with absolute ethanol with sonification to remove residual DMF after the immobilization.

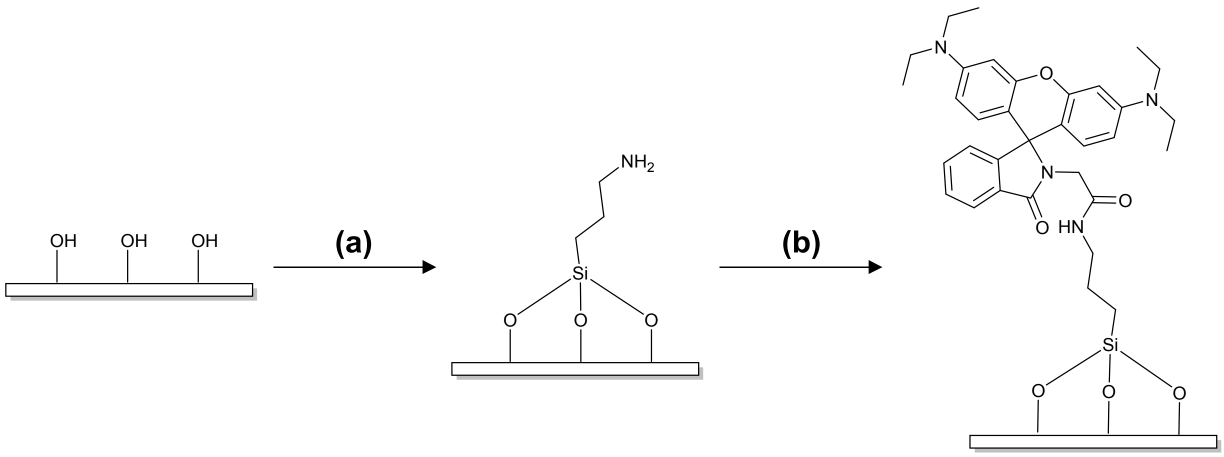

Scheme S1. Synthetic procedure to functionalize RhGly at the glass surface. (a) APTES, (b) RhGly, HATU, DIPEA.

Fluorescence lifetimes were measured using a MicroTime 200 confocal microscope (PicoQuant GbmH) with Olympus IX-71 microscope body and a 100 × 1.4 N.A. objective (UplanSApo, Olympus). Excitation light was generated by a Supercontinuum Laser (SuperK Extreme Supercontinuum, NKT Photonics) at 560 nm. Emission from the sample was detected using time-resolved single photon counting with a PDM Series detector (PicoQuant GmbH). Decay time traces were processed and fitted with SymPhoTime64 with reconvolution method.

Steady-state emission spectra were measured by means of an EMCCD (PhotonMAX, Princeton Instruments/Acton) camera attached to a spectrometer (Spectra Pro-150, Acton Research Instruments). The wavelength of the spectrograph was calibrated using the NKT Supercontinuum Laser.

All friction experiments were performed on a home built widefield/TIRFM microscope setup using a Ti:Sapphire Laser (Chameleon Ultra, Coherent; P~2400 mW @ 830 nm; 80 MHz). The laser light (829 nm) passes through an optical parametric oscillator (Mira OPO PP-Automatic, Coherent) to generate excitation at 560 nm. Contact was applied using a rheometer (DSR 301, Anton Paar) mounted on the scanning stage of the microscope. To obtain the contact images, the microscope is first focused after making a contact. Then the polymer bead is lifted, the sample is moved to a fresh area, the contact is made on the new area and data recording started. During all the experiments the pixel size is fixed at 129 nm/pixel.

PMMA (diameter = 1.45 mm) and PS (d = 1.55 mm) polymer beads (Cospheric, Santa, United States) were roughened with 240 grits sandpaper in a 50 mL centrifuge tube with a lab shaker (VXR basic Vibrax, IKA) for 16 hours. The contact areas A_r were determined by counting the contact pixels from the microscopy images applying Otsu thresholding to create a binary image [2]. Movies were created using ImageJ.

The average intensity <I> in Figure 2 in the main text is proportional to the fluorescence quantum yield of surface bound RhGly, which may depend on the medium (dry contact, isopropanol, silicon oil). The quantum yields are not

known, but we measured the fluorescence decay times, which are proportional to the quantum yields. In Figure S2B fluorescence decay curves of the functionalized RhGly in different contact environments are shown, and they are almost the same for all lubricants. We confirm this by measuring 5 independent points within the contact area, and the standard deviation on the decay time is < 1% within the same lubricated environment. The black circles in Fig 2C in the main text represent </> rescaled by dividing by the lifetime so that they correspond quantitatively to the number of active molecules under shear.

The Young's moduli of the polymer beads are derived from the contact images of a smooth polymer bead pressed on a flat RhGly-functionalized glass surface with varying the normal force at the interface, using Hertz's contact model (Eq. S1).

$$A_r = \pi\left(\frac{3R}{4E^*}\right)^{\frac{2}{3}} F^{\frac{2}{3}} \qquad \text{(S1)}$$

$$\frac{1}{E*} = \frac{(1-\nu_1)^2}{E_1} + \frac{(1-\nu_2)^2}{E_2} \qquad \text{(S2)}$$

In Equation. S1 R and F are the radius of the polymer beads, and the normal force, respectively. E^* is defined according to Equation. S2, in which ν is a Poisson's ratio, and E a Young's modulus. The subscripts 1 and 2 refer to the two materials in contact. The properties of the glass are taken from the previous literature [3]. The Young's modulus of PMMA that provides the best fit to the experimental results was used in the BEM simulation [4].

Simulation of the shear stress and normal stress was performed using a boundary element model (BEM) which couples the vertical and lateral deformation by using a linear elastic model [5–7]. The polymer bead surface geometry (Figure S6) was obtained using a Keyence optical profilometer. Comparing the simulation to the fluorescence images, we applied a rigid translation to the experimental stack to super-impose two independent measurements in ImageJ [8].

Supplementary Figures and Tables

Figure S1 demonstrates the fluorescence image (Emission spectra in Figure 1) of RhGly functionalized coverslips measured by abovementioned confocal setup. At the ambient environment, the probe is in the OFF state where the excitation at 560 nm does not excite the molecules. Switching of the probe can be achieved by adding 1% acetic acid in ethanol which protonates the spirolactam to the ON state. After removing the acid by blowing the surface with nitrogen, the active molecules immediately switch back to the spirolactam form.

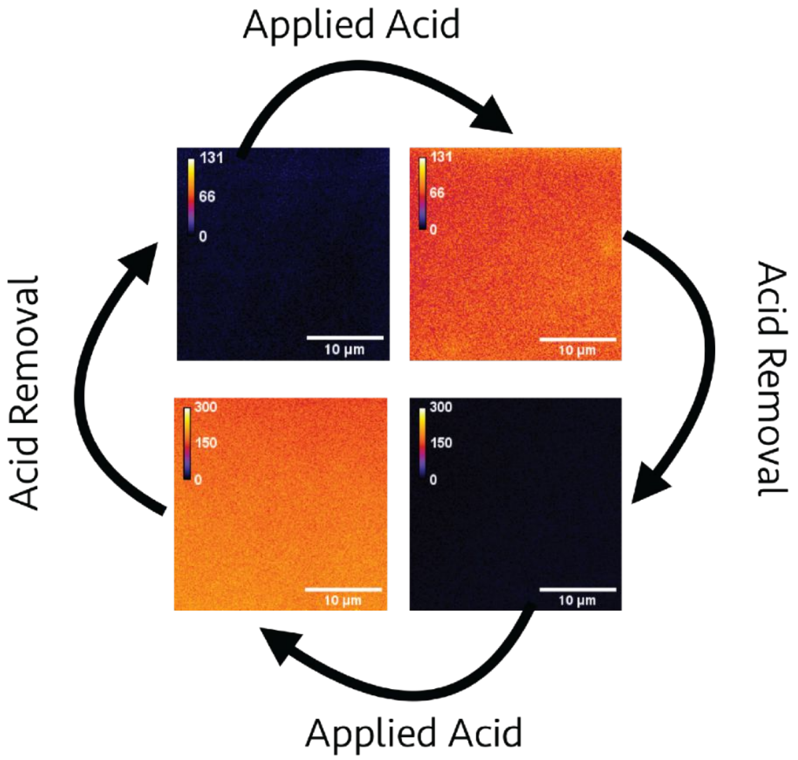

Figure S1 Fluorescence images of the RhGly functionalized coverslips. The switching of the probes can be achieved via acidification with acetic acid, and the back-switching occurs upon acid removal (evaporation).

Figure S2 shows the contact pressure (F_n/A_r) and shear stress (F_f/A_r) during the sliding at the dry and lubricated interfaces. It shows that the pressure is the same during the dynamic sliding for three experiments, and the shear stress is reduced by lubrication.

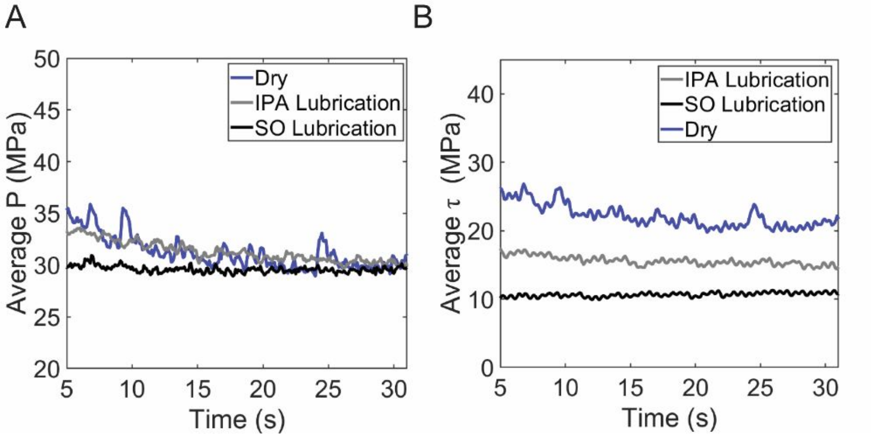

Figure S2 Average pressure *P* and average shear stress τ during the sliding experiment, where the interfaces are lubricated by isopropanol alcohol (IPA) and silicone oil (SO), comparing to the "dry" interface.

In Figure S3, we record the fluorescence intensity upon the contact application. The interfaces are selectively lubricated by the two lubricants. It is found that the fluorescence intensity decreases as the lubricating ability is larger, because lubrication will decrease the lateral force at the interface at the contact. Furthermore, the fluorescence lifetimes are measured in the three different conditions (Figure S3B). This shows that the lubricants do not affect the lifetime much, and since the radiative decay rate constant is unlikely to change much, we conclude that the intrinsic intensity (proportional to the fluorescence quantum yield) is also practically unchanged. Therefore, we can confirm that the higher luminescence intensity at the less lubricated interfaces is due to more probes being switched by lateral force.

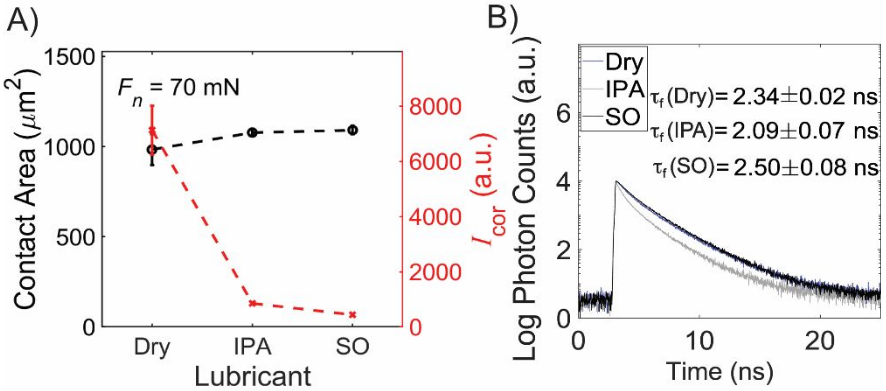

Figure S3 (A) Real contact area and average fluorescence intensity upon the application of normal force to a bead on the RhGly coated cover slips, comparing interfaces lubricated by isopropanol (IPA), silicone oil (SO) to the dry contact. The contact area does not depend on the lubricant ability (SO>IPA>dry). The intensity, however, is smaller for the lubricated contacts because the shear force is smaller. (B) Fluorescence intensity time profiles for lubricants silicone oil (SO) and isopropanol (IPA) versus no lubricant ("dry") revealing only small differences in the quantum yield of fluorescence.

We found that in Figure 2A that the shear stress is strongly correlated with the intensity within the real contact area, and thus, the fluorescence at the single pixel level can reflect the local shear stress. To approximate the validity of Coulomb's law during the sliding, in Figure S4 we calculate the radially averaged fluorescence intensity with respect to the contact center; it shows that during the sliding, the luminescence is higher closer to the center of the contact. Comparing it to Hertzian pressure at the identical contact condition, the two curves show a clear resemblance, suggesting that the local shear stress is proportional to pressure. The ratio of these two parameters is then defined as the macroscopic friction coefficient, i.e. $\tau(r)/p(r) = \mu$, which can be applied on both microscopic and macroscopic scale during the dynamic event.

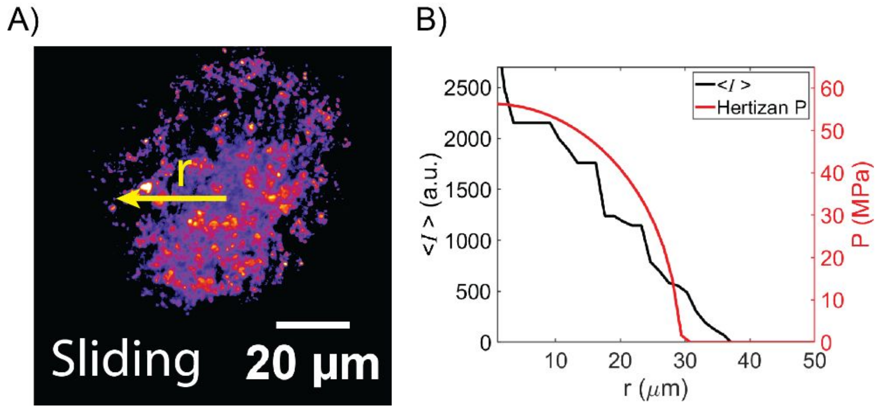

Figure S4 (A) Fluorescence images during the sliding (t = 7.1s in Figure 1A). The distribution of fluorescence intensity at single pixel level represents the local shear stress within A_r. (B) Radially averaged fluorescence intensity (black curve) with respect to the center of contact defined as the mean value of all contact points (Yellow line in A), which has a higher value close to the center of contact. The trend of the distribution is not changing during sliding. The intensity distribution shows the resemblance to the Hertzian pressure at the same F_n, indicating that during the dynamical sliding, the local shear stress is proportional to local pressure, and Coulomb's law approximately holds at the microscopic level during the sliding.

In Figure S5, we show the contact area of the smooth PMMA bead under different normal force. Fitting the contact area with Equation S1, Young's modulus of the polymer can be acquired. The modulus is used as a parameter in the BEM simulation.

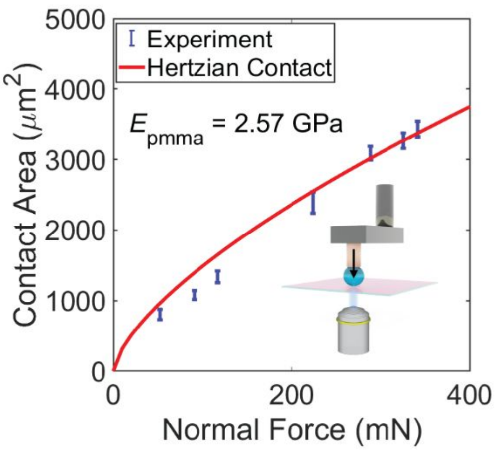

Figure S5 Measurement of Young's modulus of a smooth PMMA bead at the contact interface with an RhGly functionalized coverslip. The contact area measured at different normal forces was fitted with Equation S1 by choosing the E* to provide the best fit. The E of the PMMA can then be acquired from Equation S2 with known Poisson's ratios and the modulus of the glass.

In Figure S6, to quantify the shear stress in the fluorescence images, the topography of the bead surface was recorded using the optical profilometer before we performed a sliding experiment. The measured topography in Figure S6 was later used in the frictional simulation to obtain the local shear stress in contact.

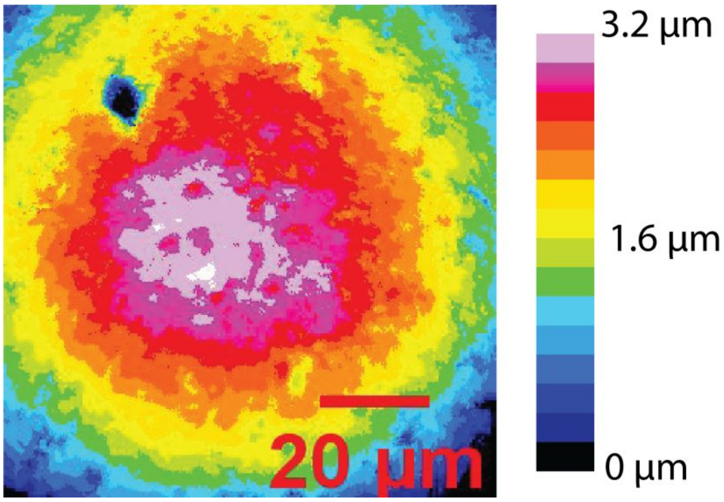

Figure S6 Topography of PMMA surface recorded by the optical profilometer.

In Figure S7, the shear stress and average fluorescence intensity are plotted during the sliding experiments of Figure 3, and Figure 4. In Figure S8, we plot the ratio of shear stress to pressure before the macro-sliding of the single asperity contact with the analytical Mindlin solution [10]. It shows resemblance to the result found in our experiment.

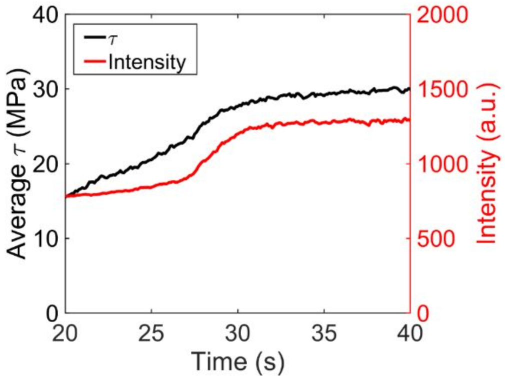

Figure S7 The force time curve and the average fluorescence intensity during the friction experiment for results showing in Figure 3 and Figure 4 in the main text. The polymer bead was not roughened during in this experiment. The macro-slip was found to occur at 32 s.

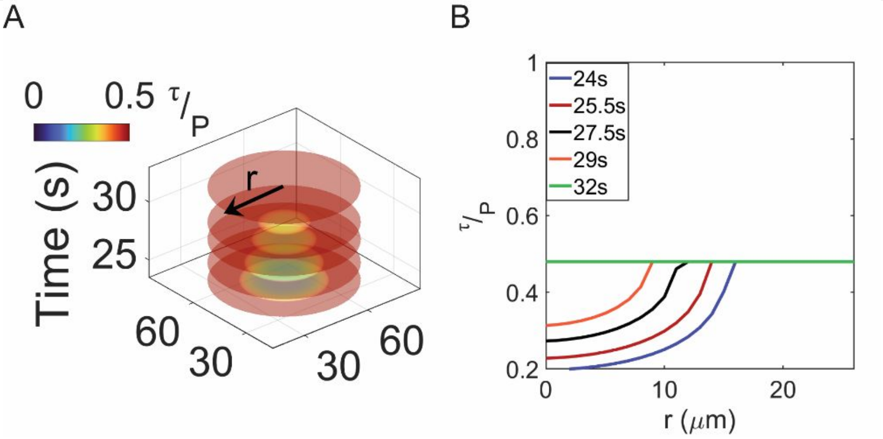

Figure S8 (A) The Mindlin solution, which predicts the shear stress during the pre-slip of a sphere-on-flat contact, showing the ratio of shear stress to pressure with respect to the contact center of a smooth sphere during the pre-sliding. (B) The radially averaged ratio of shear stress to pressure. The solution assumes that the friction coefficient is the same on macro- and microscale, which is consistent with our experimental result.

In Figure S9, we measured the returning rate of the active RhGly back to spirolactam state. To do so, we imposed a shear force at the contact interface and measured the intensity after the shear force was lifted. The returning lifetime without the contact is found to be ~230 ms and inside the contact is ~500 ms regardless of the contact pressure. During all the experiments, we always observed the returning of the probe back to the non-active state.

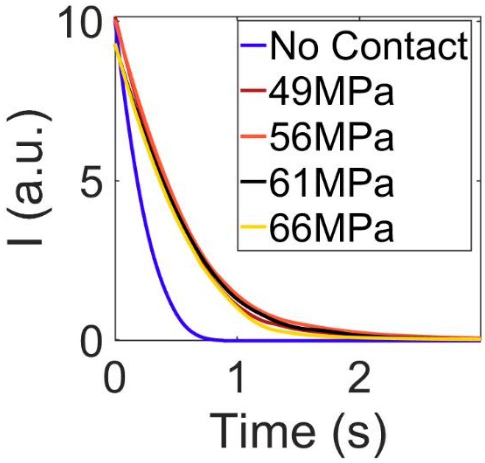

Figure S9 The back-switching rate of active RhGly back to the spirolactam state at different contact pressures.The input parameters for the BEM simulation can be found in Table S1. After inputting the parameters, a rigid displacement was applied in the simulation. We gradually increase this displacement, effectively increasing the friction force at the interface. The onset of sliding occurs when the displacement is equal to 700 nm. The ratio of the in-plane stress to the vertical stress was calculated. We averaged this ratio radially with respect to the contact center which was calculated as the mean of all the contact points. The simulated results show that the slipping zone migrates toward the center of the contact.

Table S1. Input parameters for BEM simulation.

E_{glass} (GPa)	E_{pmma} (GPa)	ν (glass)	ν (PMMA)	F_n (mN)	μ
72	2.57	0.23	0.34	105	0.48

Supplementary Movies

Movie S1.
Time series of fluorescence intensity images corresponding to Figure 2. The color scale represents intensity in arbitrary units.

Movie S2.
Quantitative images of the shear stress τ (as in figure 3A) during the sliding shown in Figure S7, starting at $t \approx 29$ s. The color scale represents τ in MPa.

Movie S3.
Animated images of the ratio of shear stress to pressure during the transition shown in Figure 4. The light color corresponds to areas where τ/P reaches the macroscopic friction coefficient $\mu_{macro} \approx 0.5$.

References

(1) Ye, Z.; Yu, H.; Yang, W.; Zheng, Y.; Li, N.; Bian, H.; Wang, Z.; Liu, Q.; Song, Y.; Zhang, M.; Xiao, Y. Strategy to Lengthen the On-Time of Photochromic Rhodamine Spirolactam for Super-Resolution Photoactivated Localization Microscopy. *J. Am. Chem. Soc.* **2019**, *141* (16), 6527–6536. https://doi.org/10.1021/jacs.8b11369.

(2) Otsu, N. A Threshold Selection Method from Gray-Level Histograms. *IEEE Trans. Syst. Man. Cybern.* **1979**, *C* (1), 62–66.

(3) Weber, B.; Suhina, T.; Junge, T.; Pastewka, L.; Brouwer, A. M.; Bonn, D. Molecular Probes Reveal Deviations from Amontons' Law in Multi-Asperity Frictional Contacts. *Nat. Commun.* **2018**, *9* (1), 888-1–7. https://doi.org/10.1038/s41467-018-02981-y.

(4) Suhina, T.; Weber, B.; Carpentier, C. E.; Lorincz, K.; Schall, P.; Bonn, D.; Brouwer, A. M. Fluorescence Microscopy Visualization of Contacts between Objects. *Angew. Chem., Int. Ed.* **2015**, *54* (12), 3688–3691. https://doi.org/10.1002/anie.201410240.

(5) Bazrafshan, M.; de Rooij, M. B.; Schipper, D. J. The Effect of Adhesion and Roughness on Friction Hysteresis Loops. *Int. J. Mech. Sci.* **2019**, *155*, 9–18. https://doi.org/10.1016/j.ijmecsci.2019.02.027.

(6) Bazrafshan, M.; de Rooij, M. B.; de Vries, E. G.; Schipper, D. J. Evaluation of Pre-Sliding Behavior at a Rough Interface: Modeling and Experiment. *J. Appl. Mech.* **2020**, *87* (4) 041006.

(7) Bazrafshan, M.; de Rooij, M. B.; Schipper, D. J. On the Role of Adhesion and Roughness in Stick-Slip Transition at the Contact of Two Bodies: A Numerical Study. *Tribol. Int.* **2018**, *121*, 381–388. https://doi.org/10.1016/j.triboint.2018.02.004.

(8) Abràmoff, M. D.; Magalhães, P. J.; Ram, S. J. Image Processing with ImageJ. *Biophotonics Int.* **2004**, *11* (7), 36–41. https://doi.org/10.1201/9781420005615.ax4.

(9) Mindlin, R. D.; Deresiewicz, H. Elastic Spheres in Contact Under Varying Oblique Forces. *J. Appl. Mech.* **1953**, *20* (3), 327–344. https://doi.org/10.1115/1.4010702.
